# Assessment of Hypertensive Patients’ Complex Metabolic Status Using Data Mining Methods

**DOI:** 10.3390/jcdd10080345

**Published:** 2023-08-13

**Authors:** Beáta Kovács, Ákos Németh, Bálint Daróczy, Zsolt Karányi, László Maroda, Ágnes Diószegi, Mariann Harangi, Dénes Páll

**Affiliations:** 1 Division of Metabolic Diseases, Department of Internal Medicine, Faculty of Medicine, University of Debrecen, H-4032 Debrecen, Hungary; kovibea01@gmail.com (B.K.); akos.nemeth@gmail.com (Á.N.); karanyi.zsolt@med.unideb.hu (Z.K.); dioszegi.agnes87@gmail.com (Á.D.); harangi.mariann@med.unideb.hu (M.H.); 2 Institute for Computer Science and Control (SZTAKI), Hungarian Research Network, H-1111 Budapest, Hungary; daroczyb@ilab.sztaki.hu; 3 Department of Mathematical Engineering (INMA/ICTEAM), Université Catholique de Louvain, 1348 Louvain-la-Neuve, Belgium; 4 Department of Medical Clinical Pharmacology, Faculty of Medicine, University of Debrecen, H-4032 Debrecen, Hungary; maroda.laszlo@unideb.hu; 5 Institute of Health Studies, Faculty of Health Sciences, University of Debrecen, H-4032 Debrecen, Hungary

**Keywords:** hypertension, hyperuricemia, dyslipidaemia, metabolic parameters, obesity, prevalence, statin, data mining, cardiometabolic

## Abstract

Cardiovascular diseases are among the leading causes of mortality worldwide. Hypertension is a preventable risk factor leading to major cardiovascular events. We have not found a comprehensive study investigating Central and Eastern European hypertensive patients’ complex metabolic status. Therefore, our goal was to calculate the prevalence of hypertension and associated metabolic abnormalities using data-mining methods in our region. We assessed the data of adults who visited the University of Debrecen Clinical Center’s hospital (n = 937,249). The study encompassed data from a period of 20 years (2001–2021). We detected 292,561 hypertensive patients. The calculated prevalence of hypertension was altogether 32.2%. Markedly higher body mass index values were found in hypertensive patients as compared to non-hypertensives. Significantly higher triglyceride and lower HDL-C levels were found in adults from 18 to 80 years old. Furthermore, significantly higher serum glucose and uric acid levels were measured in hypertensive subjects. Our study confirms that the calculated prevalence of hypertension is akin to international findings and highlights the extensive association of metabolic alterations. These findings emphasize the role of early recognition and immediate treatment of cardiometabolic abnormalities to improve the quality of life and life expectancy of hypertensive patients.

## 1. Introduction

Hypertension is one of the most potent preventable risk factors for premature death and cardiovascular disorder [1]. Approximately 54% of stroke, 47% of coronary heart disease, 75% of hypertensive disease and 25% of other cardiovascular disease cases have been shown to be attributable to high blood pressure [2]. Global surveys show that rates of raised systolic blood pressure have increased gradually over the last four decades particularly in low- and middle-income countries with the knock-on effects of increased disability-adjusted years of life and deaths attributable to hypertension [3]. 

Based on an analysis of data from 135 population-based studies that included 968,419 adults from 90 countries, in 2010, the global age-standardised prevalence of hypertension was 31.1%, which was slightly higher in men (31.9%) than in women (30.1%). The highest prevalence was in Eastern Europe and Central Asia (39.0%) [4]. The Prospective Urban Rural Epidemiology (PURE) study reported that between 2003 and 2009, in 153,996 adults aged 35–70 years from 628 rural and urban communities in economically and geographically diverse countries, 40.8% of subjects had hypertension with a higher prevalence in men (41.4%) than in women (37.7%) [5]. Besides gender, non-modifiable risk factors of hypertension include age, ethnicity, and genetics, while modifiable risk factors are obesity and overweight, salt, potassium or saturated fat excess, alcohol intake, stress, physical inactivity, and socio-economic status.

Cardiovascular diseases are at the top of Hungarian mortality lists. It must be noted that the mortality rate is twice as high as the European Union average [6]. Therefore, the identification of patients with hypertension is of utmost importance. According to data from the Hungarian Hypertension Registry and the Hungarian Central Statistical Office, in 2011, the estimated prevalence of hypertension in Hungary was 35%, the male/female ratio was 47/53% [7]. Hungary joined the May Measurement Month (MMM) programme, which is an annual global initiative aimed at raising awareness of high blood pressure. Out of 2766 Hungarian individuals screened in MMM, 1286 participants (46.5%) had hypertension [8]. According to the database of the European Society of Cardiology, in 2021, the prevalence of hypertension in Hungary was 30%, accompanied by a 26.4% prevalence of obesity and a 3275/100,000 prevalence of ischemic heart disease [6]. 

Previously the metabolic status of Central and Eastern European hypertensive adult patients has not been thoroughly explored. Therefore, we aimed to identify hypertension and its associated metabolic abnormalities and analyse a relatively large adult population in our region by evaluating a period of 20 years using data mining methods. Data mining is currently not a common approach in epidemic studies. However, the method seems appropriate to define prevalence in large patient populations. Assessment of previous and current therapeutic approaches regarding metabolic abnormalities associated with hypertension can be the base of efficient medical quality control in the future. Data mining is an excellent tool to explore the flaws and weaknesses of the current clinical practice. To our knowledge, bearing any relevant medical statistical information is a way of quality improvement. This improvement can occur in several aspects of everyday clinical practice, such as screening and therapy setup. Therefore, it is a welcome notion that we have a new and reliable statistical tool which may help us devise an accurate clinical decision-making process. We hypothesized that the prevalence of hypertension in Hungary is in tune with international data. However, due to poor health awareness in our region, we expected a relatively high prevalence of concomitant metabolic disturbances that may explain the outstanding cardiovascular morbidity and mortality in our country.

## 2. Materials and Methods

### 2.1. Screening Patients for Hypertension 

In accordance with findings in our previous studies [9,10,11,12], data mining methods directed at mass hospital data are an excellent novel tool for screening for medical diagnoses of hypertension and other health conditions. Specialists established clinical diagnoses of hypertension adhering to widespread international guidelines utilising validated blood pressure measuring devices. Diagnoses of hypertension were found in textual history data (data search directed at the phrases ‘hypertension’ and synonyms) and diagnosis codes for hypertension (International Statistical Classification of Diseases and Related Health Problems 9th and 10th Revision, WHO) which were also found in the source records.

The University of Debrecen Clinical Center provided access to anonymous medical records compiled in the north-eastern region of Hungary for the purpose of software development, such as full medical records from the clinical centre from a period of 1 January 2001 to 31 December 2021. The collaborative efforts of our research teams consisted of a data mining project. Clinical records data extracts were subjected to a complex procedure of anonymization to protect patients’ private data and the separation of tables containing specific case and patient data from the identified ones. We compiled a totalised and integrated data source from isolated laboratory cases, anamneses, national diagnosis codes, and patient statistical data. By serialising and buffering the data we warded off certain typical problems arising from dealing with enormous data sources. During pre-processing, we processed textual information through parsing and stemming (http://hunspell.github.io/ (accessed on 21 June 2022)) and bag-of-words (BOW) modelling. Phrases were ranked by the “Term Frequency/Inverse Document Frequency” (TF-IDF) method [13] and word2vec (W2V) modelling was performed in Keras (https://keras.io/ (accessed on 21 June 2022)) to pinpoint key role phrases [14]. The establishment of BOW models involves outlining documents as histograms of recurrent phrases. These models, however, do not emphasise sequentiality, and therefore, representations are vigorous and invariant where the original documents would have deviating elements of sequences. We also established a phrase list using professional vocabulary and utilised string-matching algorithms to avoid misspellings.

The extracted and processed data also contains anamneses. These have not been processed in any way whatsoever and thus pre-processing methods are necessary such as text extraction and regular terminology-based content identification. The resulting data are a finite set of phrases with values for a number of occurrences per document and for previous medical check-ups. Further details of the data mining process are summarized in our previous papers [9,12]. The main steps of the data processing are demonstrated in Supplementary Figure S1. 

The project meets the requirements of the Declaration of Helsinki. The protocol was approved by local and regional ethical committees (DE RKEB/IKEB 5028-2018, date of approval: 25 June 2018; TUKEB 44549-2/2018/EKU, date of approval: 29 August 2018).

### 2.2. Determining Cardiovascular Risk factors in Laboratory Environment

Since we had datasets with various data structures, it was necessary to establish joint representation for facile statistical analysis. This kind of structure ensures the detection of specific ‘attributes’. Hypertension might appear in the textual data under different phrases. The challenge of data cleansing had to be surmounted too by completing missing or corrupt data fragments, and individual types of corrupted data were treated in different ways. The astounding magnitude of data necessitated further serialising and streaming methods to be developed for the optimisation of the final query engine, which now has the skill to manage partial data and identify attributes based on deduction. Word embeddings in Hungarian were trained on traditional corpora, which presupposed the compilation of a language model from the available text. Identifications and high-probability cases were based on the language model that showed such to be the result of data from laboratory measurements.

### 2.3. Laboratory Analysis

Routine laboratory parameters such as glucose, uric acid, triglyceride, alanine transferase (ALT), aspartate aminotransferase (ALT), and gamma-glutamyl transferase (GGT) from fresh sera were measured with the help of a Cobas c501 and Cobas c600 analysers (from Roche Ltd., Mannheim, Germany) at the Laboratory Medicine Unit, Clinical Centre, University of Debrecen. Total cholesterol level measurements were performed through enzymatic and colorimetric tests (cholesterol oxidase-p-aminophenazone—GPOD-PAP; Modular P-800 analyser; Roche/Hitachi). Measuring high-density lipoprotein cholesterol (HDL-C) and low-density lipoprotein cholesterol (LDL-C) levels used a homogenous enzymatic and colorimetric assay (Roche HDL-C as well as the third generation for HDL-C and Roche LDL-C plus second generation for LDL-C). Immunoturbidimetric assays (Tina-quant apolipoprotein A-I ver. 2 and Tina-quant apolipoprotein B ver. 2, respectively) were used to measure apolipoprotein A1 (ApoA1) and apolipoprotein B100 (ApoB100) levels. Tests were performed as per the manufacturer’s recommendations.

### 2.4. Statistical Analysis

We used anonymous patient data obtained from the University of Debrecen Clinical Center’s computerised clinical system. Access to the data source in HL7 format was granted by our partner company, who had partially cleaned and pre-processed the data in an effort to use them in data mining and machine learning projects. By leveraging the database at the start, we avoided potential system errors resulting from the original data-recording method, spanning a 20-year period, containing the totality of the clinical centre patient record database including all textual, diagnostic and laboratory data. See our previous papers for a detailed description of the statistical analysis [9,12]. Briefly, the extraction of data was performed by queries from the PostgreSQL 13.x database. The resulting large-size text files were then utilised as initial databases for statistical analysis later on. We conducted statistical analysis by deploying Python-supported data-mining packages. We used of Python 3.8, IPython 7.29, Cython 0.29, Pandas 0.23 and Numpy 1.22 under Conda 4.10 environment with Dask for data cleaning and processing phases. We deployed machine learning to refine data selection and perform deep textual analysis leveraging SciKit-Learn 1.0 and Pytorch 1.09. We conducted unpaired *t*-tests for statistical significance analysis and maintained a significance level of 95%. Statistical figures were compiled using the MatPlotLib 3.5 package.

## 3. Results

### 3.1. Identification of Patients with Hypertension

**Figure 1:**
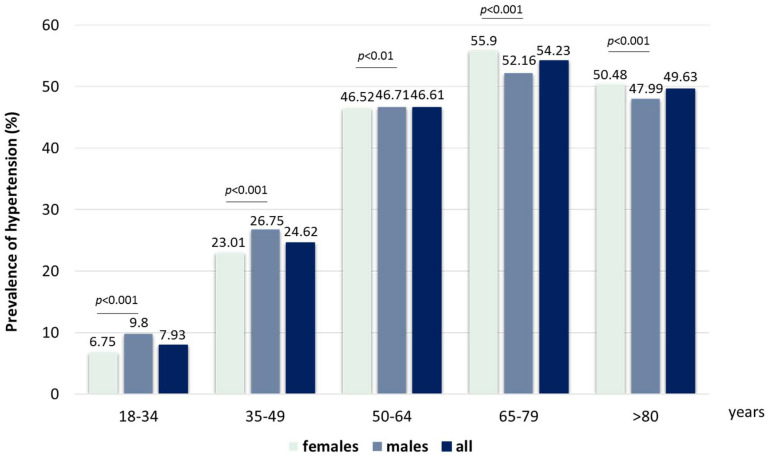
Prevalence of hypertension in different age and gender groups.

We evaluated data from 937,249 adults over 18 years old who visited the University of Debrecen Clinical Center’s hospital throughout the 20-year study period (males/females 42.9/57.1%). We identified a total of 292,561 adults with hypertension (male/female: 45.4/54.6%), with a 32.2% calculated prevalence of hypertension in the whole study population and a higher prevalence in males (33%) compared to females (29.9%). We found an increasing prevalence in consecutive age groups both in males and females up to the age of 80. The ratio of males with hypertension was higher from 18 to 65 years of age, while it was lower above 65 years of age (Figure 1).

### 3.2. Body Mass Index

**Figure 2:**
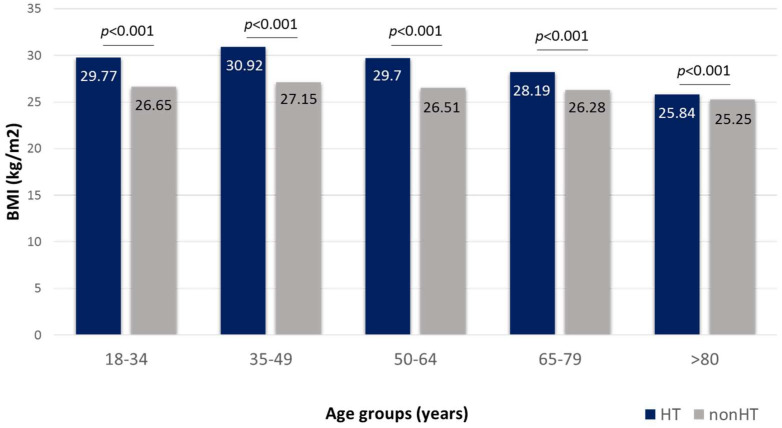
Average body mass index (BMI) values in subjects with (HT) and without (nonHT) hypertension in different age groups.

Markedly higher body mass index (BMI) values were found in hypertensive patients as compared to non-hypertensives in the entire study population (29.5 ± 6.3 vs. 26.57 ± 5.6 kg/m^2^, *p* < 0.001) and in all age groups up to 80 years of age, with a highest mean value in the 35–49-year-old age group (30.92 ± 7.3 kg/m^2^) (Figure 2). 

### 3.3. Lipid Parameters

**Figure 3:**
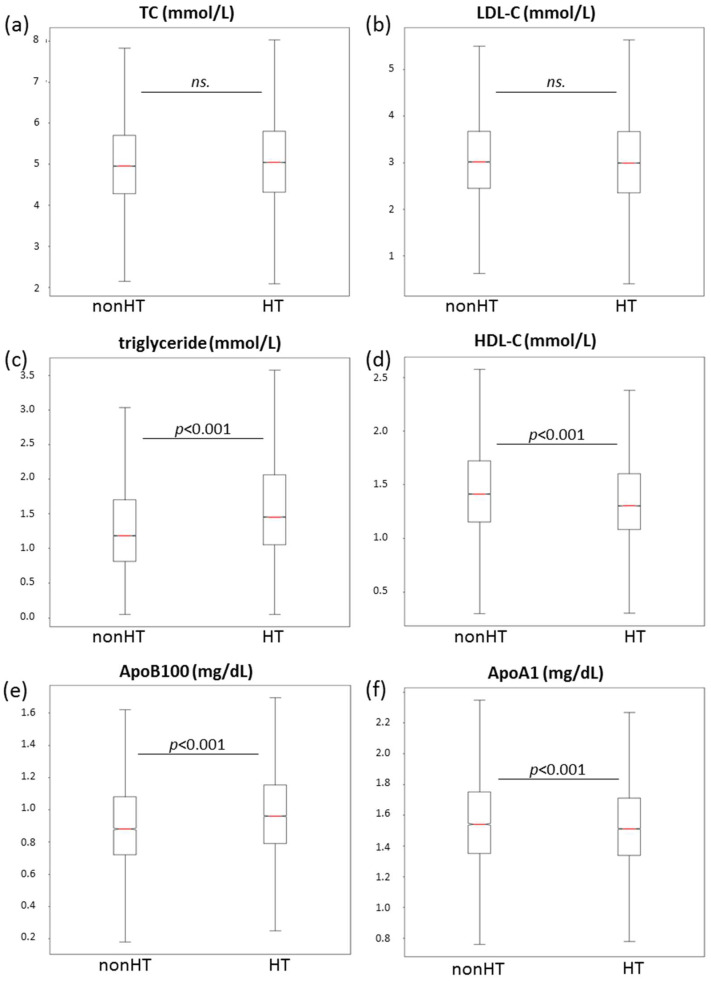
Boxplots and whiskers of (**a**) serum TC, (**b**) LDL-C, (**c**) triglyceride, (**d**) HDL-C, (**e**) ApoB100 and (**f**) ApoA1 in subjects with (HT) and without (nonHT) hypertension in the whole study population. The length of the box represents the interquartile range (IQR), the horizontal line in the box interior represents the median, the whiskers represent the 1.5 IQR of the 25th quartile or 1.5 IQR of the 75th quartile. ApoA1: apolipoprotein A1; ApoB: apolipoprotein B100; HDL-c: high-density lipoprotein; LDL-C: low-density lipoprotein; ns: non-significant; TC: total cholesterol.

Interestingly, we could not find significant differences in total cholesterol and LDL-C levels in the entire patient population (5.1 ± 1.2 vs. 5.0 ± 1.2 mmol/L and 3.1 ± 0.99 vs. 3.1 ± 1.0 mmol/L, respectively) (Figure 3a,b). 

Moreover, significantly lower serum total cholesterol and LDL-C levels were found in age groups 35–49 years, 50–64 years, and 65–79 years in hypertensives as compared to normotensives. The serum total cholesterol level was significantly higher in the hypertensive >80-year-old age group compared to normotensives (Table 1). 

A significantly higher triglyceride level was found in hypertensive adults compared to non-hypertensives in the entire study population (1.46(1.06–2.07) vs. 1.19 (0.82–1.73 mmol/L, *p* < 0.001) (Figure 3c). Regarding the consecutive age groups, serum triglyceride levels were higher from 18 to 80 years of age (Table 1). Serum HDL-C levels were significantly lower in hypertensive subjects compared to normotensives in the entire study population (1.36 ± 0.4 vs. 1.46 ± 0.6 mmol/L, *p* < 0.001) (Figure 3d). Furthermore, lower serum HDL levels were detected in all age groups (Table 1). 

A significantly higher ApoB100 level was found in hypertensive adults compared to non-hypertensives in the entire study population (0.99 ± 0.3 vs. 0.93 ± 0.33 g/L, *p* < 0.001, respectively) (Figure 3e). In addition, higher ApoB100 levels were found in most age groups (from 18 to 80 years of age) (Table 1). Serum ApoA1 levels were significantly lower in hypertensive subjects compared to normotensives in the whole study population (Figure 3f). Similarly, we found lower serum ApoA1 levels in all age groups except for the 65–79-year-old age group (Table 1). 

### 3.4. Identification of Statin Users

**Figure 4:**
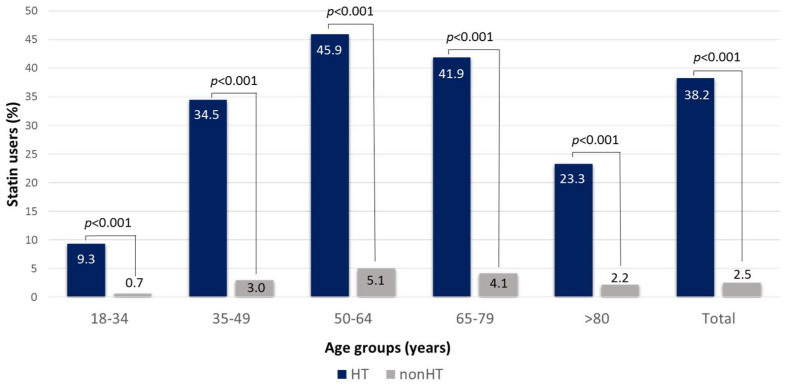
Statin users in subjects with and without hypertension in different age groups in the total study population.

Based on our data, the ratios of statin users in hypertensive subjects as compared to normotensives were markedly higher in all age groups (Figure 4).

### 3.5. Serum Glucose, Haemoglobin A1c and Uric Acid Levels, Renal and Liver Function

Significantly higher serum glucose levels (5.8 (5.2–6.9) vs. 4.7 (4.6–5.8) mmol/L, *p* < 0.001) were found in subjects with hypertension compared with the non-hypertensive subjects in the entire study population, as well as in all age groups (Table 1). HbA1c levels were also significantly higher in hypertensives in all patient groups except for the 65–79-year-old age group (Table 1). Furthermore, serum uric acid levels were significantly higher (321 ± 96.6 vs. 278 ± 88.2 μmol/L, *p* < 0.001) in subjects with hypertension compared with non-hypertensive subjects in the entire study population, as well as in all age groups ***(***Table 1). Moreover, significantly higher urea and creatinine levels were found in the entire hypertensive group compared to normotensives (5.78 (4.6–7.5) vs. 4.6 (3.7–5.7) mmol/L, *p* < 0.001 and 76 (64–92) vs. 69 (59–81) μmol/L, *p* < 0.001, respectively) and in most age groups (Table 1). AST activity was significantly higher in HT patients compared to non-HT subjects only in the 18–34-year-old age group. ALT was significantly higher in most age groups, while GGT was higher in HT patients compared to normotensives in all age groups (Table 1). 

### 3.6. Concomitant Diseases

**Figure 5:**
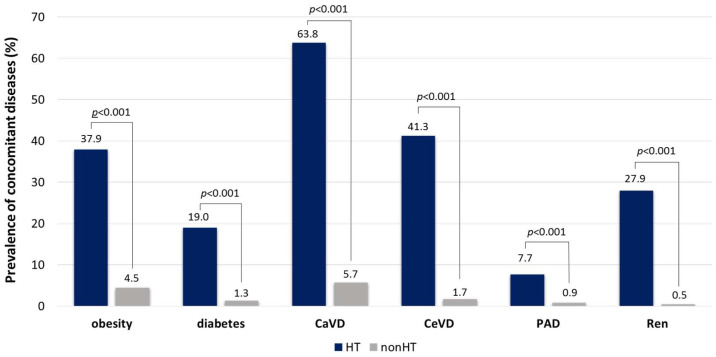
Prevalence of concomitant diseases in subjects with (HT) and without (non-HT) hypertension. CaVD: cardiovascular disease; CeVD: cerebrovascular disease, PAD: peripheral arterial disease, Ren: renal disease.

The prevalence of obesity, diabetes, and renal diseases was markedly higher in hypertensive patients compared to non-hypertensives. Furthermore, the prevalence of atherosclerotic vascular complications including cardiovascular, cerebrovascular, and peripheral artery diseases were significantly higher in hypertensive subjects as compared to normotensives (Figure 5).

In HT patients the highest obesity prevalence was found in the 35–49 and 51–65 y age groups (37.2 and 37%), the lowest was in the >80 y group (21.4%). The diabetes prevalence was the highest in the 65–74 y age group (22.8%), while the lowest in the 18–34 y age group (5.1%). The prevalence of CaVD gradually increased in the consecutive age groups from 17.8 to 53.4%. The prevalence of CeVD dramatically increased in elderly patients (26.9 and 29.8% in 65–79 y and >80 y, respectively). The prevalence values of PAD were relatively low in all age groups and varied between 2.2 and 6.2%. Interestingly, the highest prevalence was found in the 35–49 y age group. The prevalence of renal diseases gradually increased from 4.4 to 24.8% in the consecutive age groups. Similar tendencies have been found in the non-HT population, but the prevalence values were approximately ten times lower compared to HT patients (Supplementary Figure S2).

### 3.7. Laboratory Parameters of the Study Population Based on the Grade of Hypertension

Hypertensive patients were divided into three groups based on the grade of hypertension. Grades were defined according to the 2018 ESC/ESH Guidelines for the management of arterial hypertension [15]. Grade 1 was defined as systolic blood pressure (SBP) 140–159 mmHg and/or diastolic blood pressure (DBP) 90–99 mmHg; Grade 2 was defined as SBP 160–179 mmHg and/or DBP 100–109 mmHg; Grade 3 was defined as SBP ≥ 180 mmHg and/or DBP ≥ 110 mmHg. Several metabolic parameters including triglyceride, glucose and uric acid levels were significantly higher in higher grades of hypertension. Renal function showed worsening in the consecutive grade categories (Table 2).

## 4. Discussion

This is the first study which explored Central and Eastern European hypertensive patients’ complex metabolic status in a large population. Our results conclusively demonstrate the clustering of cardiovascular risk factors including metabolic alterations not only in patients with known cardiovascular complications but in primary prevention as well. 

Data mining is the process of analysing and managing data from a large pool of information, which leads to the summarisation of the data to obtain an insight into large databases seeking unknown patterns, classifications, clustering, and relationships in the dataset [16]. Advances in the realm of computational information have allowed the development of new methods and tools to analyse large quantities of data. Data mining has made use of medical data for identifying patterns and designing predictive models to help physicians in decision-making and the treatment of patients. Furthermore, data mining has been successfully applied to discovering and establishing relationship models to display the connection between health parameters and diseases including hypertension, hyperlipidaemia, and metabolic syndrome [12,16]. It must be noted that data mining is not a superior method compared to epidemiology studies, but rather a complementary option to gain more information from massive data. We investigated a similarly large patient population with the help of data mining methods. That study encompassed a 15-year period [12]. Our present research is further evidence of the benefit of data-mining strategies in everyday clinical research to determine prevalence data and explore associations with metabolic alterations. Our data demonstrate that the calculated prevalence of hypertension is akin to international findings and in line with some previous data found in Hungarian registries [4,6,7].

The relationship between obesity and hypertension is well-established. Epidemiological studies have consistently reported a direct relationship between BMI and blood pressure that is continuous and almost linear, with no evidence of a threshold [17,18]. Higher BMI and waist circumference values were found in hypertensive females and males compared to normotensives in previous studies [19]. Our findings also support the above-mentioned observations. 

Hypertension is often associated with hyperlipidaemia [20]. In particular, high triglyceride and low HDL-C levels are dominant players in atherogenic dyslipidaemia [21]. This pathological condition is mainly associated with overweight, obesity and carbohydrate metabolism disorder. Elevated BMI and diabetes mellitus, and therefore higher glucose and HbA1c levels, frequently occur among hypertensive patients. This may result in the development of atherogenic dyslipidaemia. Additionally, elevated BMI and diabetes mellitus frequently occur in this group. According to the literature, elevated total cholesterol and LDL-C levels are also associated with hypertension [19]. The risk of death and cardiovascular events is significantly higher in patients suffering from hypertension and dyslipidaemia than the risk of each separately. This observation can be explained by the synergistic effect between the above-mentioned disorders [22]. However, we have not found a significant difference in these latter parameters with respect to hypertensive and non-hypertensive patients. Indeed, the level of cholesterol and LDL-C was lower in hypertensive patients compared to non-hypertensive ones. The higher levels of ApoB100 containing VLDL particles (indicated by the higher triglyceride levels) might explain the higher ApoB100 levels in hypertensive patients, despite the similar LDL-C levels. Lower LDL-C levels quite possibly result from the surprisingly different rate of statin use in favour of hypertensive patients. Hypertensive patients often deal with cardiovascular comorbidities and take statins regularly, which may lead to lower total cholesterol and LDL-C levels. Moreover, due to close patient care, better adherence and perhaps also the administration of fixed-dose combination antihypertensive therapy with statin content in recent years, lipid levels tend to approach target values in hypertensives. 

Of note, total cholesterol level was higher in HT patients compared to non-HT subjects in the >80 y age group. On the one hand, statin use was very low in this age group, and we also hypothesize that the statin adherence might be lower as well, since age is a key factor in statin adherence. On the other hand, total cholesterol both in HT and non-HT patients were lower in >80 y age group compared to younger patient groups. The BMI of these elderly patients is also lower, which might be responsible for the lower total cholesterol levels. This unintentional weight loss in elderly populations is well documented in the literature. Nonmalignant diseases are more common causes, but malignancy accounts for up to one-third of cases of unintentional weight loss. Medication use and polypharmacy can interfere with the sense of taste or induce nausea as causative factors. Social factors such as isolation and financial constraints may also lead to unintentional weight loss in the elderly [23]. Furthermore, patients with higher total cholesterol may pass away at a younger age compared to those with cardiovascular diseases. 

Several studies have demonstrated an association between hyperuricemia and hypertension [24,25,26]. On the one hand, in a hypertensive state, renal blood flow decreases, which results in urate reabsorption. Hypertension chronically leads to microvascular complications, hypoxia and local tissue damage, which elevates uric acid levels. Antihypertensive agents such as thiazide diuretics may also increase serum uric acid levels by stimulating proximal tubule reabsorption [26]. On the other hand, uric acid induces vasoconstriction by activation of the renin-angiotensin system and a reduction in circulating nitric oxide. Moreover, uric acid is incorporated into vascular smooth muscle cells, resulting in cellular proliferation, which brings about secondary arteriolosclerosis that impairs pressure natriuresis, causing sodium-sensitive hypertension [27]. In line with the literature, we also found significantly higher serum uric acid levels in subjects with hypertension in our study population. 

The correlation between high blood pressure and kidney disease is well established. Hypertension is both a cause and a consequence of chronic kidney disease and relates to its progression. Several mechanisms play a part in the development of hypertension in chronic kidney disease including an increased sympathetic tone, the upregulation of the renin-angiotensin-aldosterone system, leading to salt and water retention, an increased salt sensitivity, and endothelial dysfunction leading to increased arterial stiffness [28]. Once hypertension has manifested itself, plenty of factors, including increased oxidative metabolism associated with relative renal hypoxia, may enhance the further progression of renal disease [29]. We also measured significantly higher urea and creatinine levels in the hypertensive group compared to normotensives. Moreover, we also detected a higher prevalence of renal diseases among hypertensives compared to the normotensive population. 

Obesity or overweight, insulin resistance, type 2 diabetes mellitus (T2DM), hypertension and hyperlipidaemia are frequently associated with non-alcoholic fatty liver disease (NAFLD). Elevation in AST, ALT, and GGT is a marker of hepatocyte injury and a common abnormality seen in patients with underlying liver disease including NAFLD [30]. Our results are in line with the literature; in particular, GGT levels were significantly higher in hypertensives compared to normotensives. NAFLD might be another metabolic abnormality that is associated with hypertension in the study population. 

According to the finding of a previous paper [31], we detected the worsening of metabolic status, renal and liver function in higher grades of hypertension. Our results highlight the importance of metabolic status in the severity of hypertension.

Due to these complex metabolic changes, the prevalence of atherosclerotic vascular complications was inevitably significantly higher in hypertensive subjects compared to normotensives. These differences were apparent in CaVD, which was consequently observed in every age group. The extremely high prevalence of CeVD is also noted, especially in the elderly hypertensive population. The unexpectedly low prevalence of PAD indicates the shortage of screening, which is also common worldwide, since PAD has been historically underappreciated by healthcare professionals and patients [32].

If we compare our study results with the 2021 European Society of Cardiology (ESC) cardiovascular disease statistics, in 2015 the median age-standardized prevalence of elevated blood pressure (defined as systolic blood pressure ≥ 140 mmHg and/or diastolic blood pressure ≥ 90 mmHg) among adults was 30% in our country [6]. During this 20-year study period, we identified a total of 292,561 adults with hypertension out of 937,249 hospital-goers with a 32.2% calculated prevalence of hypertension. Data for 2015 proved that the prevalence of hypertension was lower in females compared to males in all ESC member countries [6]. As per the statistics, we also detected a higher prevalence in males (33%) compared to females (29.9%). Unfortunately, we could not compare all of our results with ESC data due to different study group characterisation methods. Still, these comparable results underline the fact that data mining is a competitive and reliable method when it comes to statistical analysis.

We must admit that there are some limitations to our study. We may overestimate the prevalence of hypertension because hospital-goers are not entirely representative of the general population. That is the reason why we surveyed a large patient group. Hopefully, this comprehensive analysis may provide accurate statistical data. We attempted to assess data regarding lifestyle habits, diet, and family history, too, but unfortunately, these could not be supplied due to incoherent and incomplete documentation.

## 5. Conclusions

Our study suggests that the calculated prevalence of hypertension and its concomitant metabolic abnormalities is comparable to international findings and highlights the extensive association of metabolic alterations. Having reliable nationwide statistical data is essential in designing regional Public Health strategies and optimise resource allocation. It lays the foundations of patient screening and education and assists everyday clinical decision-making strategies. It is a long-awaited method of getting accurate statistical data regarding our patients’ base and comorbidity, metabolic state and various risk factors. Data mining is an excellent yet not-too-widespread method used to define prevalence in large patient cohorts. Using this novel way of statistical analysis could be beneficial not only for regional clinical practice but nationwide strategies as well. Moreover, it could provide an opportunity for a common Central-European decision-making and screening initiative. Based on data proving the frequent clustering of cardiometabolic risk factors in hypertension, their early recognition and immediate treatment are essential to improve the quality of life and life expectancy of patients with hypertension.

## Figures and Tables

**Table 1:** Laboratory parameters of the study population. Values are presented as mean ± standard deviation or median (lower quartile–upper quartile).

	18–34 Years		35–49 Years		50–64 Years		65–79 Years		>80 Years	
	HT	nonHT	HT	nonHT	HT	nonHT	HT	nonHT	HT	nonHT
n	22,438	260,548	54,197	165,904	109,883	125,873	90,891	76,700	18,210	18,484
TC (mmol/L)	4.80 ± 1.01	4.79 ± 1.05	**5.26 ± 1.14**	**5.28 ± 1.18**	**5.22 ± 1.19**	**5.35 ± 1.31**	**4.98 ± 1.20**	**4.99 ± 1.33**	**4.74 ± 1.20**	**4.64 ± 2.31**
LDL-C (mmol/L)	2.94 ± 0.85	2.94 ± 0.97	**3.22 ± 0.94**	**3.31 ± 0.97**	**3.11 ± 0.9**	**3.31 ± 1.07**	**2.89 ± 1.01**	**3.02 ± 1. 9**	2.72 ± 0.97	2.72 ± 1.07
TG (mmol/L)	**1.25 (0.87–1.87)**	**1.01 (0.72–1.5)**	**1.56 (1.09–2.30)**	**1.28 (0.9–1.90)**	**1.56 (1.13–2.20)**	**1.40 (1.00–1.98)**	**1.40 (1.04–1.90)**	**1.30 (0.97–1.80)**	1.20 (0.91–1.60)	1.11 (0.87–1.50)
HDL-C (mmol/l)	**1.38 ± 0.41**	**1.50 ± 0.58**	**1.34 ± 0.41**	**1.45 ± 0.45**	**1.36 ± 0.41**	**1.43 ± 0.47**	**1.37 ± 0.41**	**1.38 ± 0.45**	**1.38 ± 0.42**	**1.46 ± 0.75**
ApoB100 (g/L)	**0.88 ± 0.27**	**0.86 ± 0.86**	**1.00 ± 0.28**	**0.98 ± 0.28**	**1.02 ± 0.28**	**1.01 ± 0.29**	**0.97 ± 0.28**	**0.96 ± 0.28**	**0.93 ± 0.26**	**0.92 ± 0.27**
ApoA1 (g/L)	**1.52 ± 0.33**	**1.57 ± 0.87**	**1.52 ± 0.29**	**1.56 ± 0.38**	**1.54 ± 0.29**	**1.57 ± 0.33**	**1.54 ± 0.30**	**1.53 ± 0.32**	**1.52 ± 0.33**	**1.54 ± 0.31**
glucose (mmol/L)	**5.10 (4.7–5.6)**	**4.98 (4.6–5.4)**	**5.50 (5.0–6.3)**	**5.29 (5.3–7.0)**	**5.89 (5.3–7.0)**	**5.60 (5.0–6.4)**	**6.10 (5.4–7.3)**	**5.78 (5.1–6.9)**	**6.20 (5.5–7.3)**	**5.93 (5.2–7.1)**
HbA1c (%)	**5.4 (5.1–6.0)**	**5.3 (5.0–5.6)**	**6.0 (5.5–7.2)**	**5.8 (5.4–6.8)**	**6.4 (5.8–7.5)**	**6.1 (5,6–7.1)**	6.4 (5.8–7.5)	6.4 (5.7–7.5)	**6.3 (5.8–7.3)**	**6.0 (5.5–7.0)**
uric acid (µmol/L)	**297 ± 84.2**	**265 ± 75.5**	**308 ± 89.0**	**278 ± 87.1**	**322 ± 92.9**	**297 ± 96.7**	**333 ± 103.1**	**314 ± 112.2**	**344 ± 120.7**	**335 ± 142.5**
urea (mmol/L)	**4.30 (3.55–5.19)**	**4.13 (3.40–5.0)**	**4.89 (4.06–5.83)**	**4.65 (3.80–5.60)**	**5.70 (4.70–7.0)**	**5.33 (4.38–6.60)**	**6.90 (5.53–9.02)**	**6.50 (5.20–8.50)**	**8.20 (6.30–11.1)**	**7.90 (6.0–10.9)**
creatinine (µmol/L)	**68.3 (57.8–81.2)**	**66.0 (56.3–78.0)**	**71.0 (61.0–83.7)**	**69.0 (59.5–80.5)**	**75.0 (64.0–89.7)**	**72.0 (61.0–84.7)**	**83.0 (68.9–103.9)**	**79.3 (66.0–98.0)**	89.3 (72.0–116.0)	88.0 (70.6–114.5)
AST (U/L)	**20.7 (17.1–26.3)**	**19.0 (16.0–24.0)**	21.9 (18.0–29.0)	20.7 (17.0–26.5)	22.2 (18.2–30.0)	21.9 (18.0–29.0)	22.0 (18.0–30.3)	21.9 (17.0–30.7)	23.0 (18.0–33.0)	22.3 (17.0–34.0)
ALT (U/L)	**21.0 (15.0–34.0**	**18.0 (13.0–26.9)**	**24.5 (17.5–36.2)**	**21.0 (15.0–32.0)**	23.0 (17.3–33.0)	21.0 (15.5–31.0)	**19.7 (15–27.8)**	**19 (13.7–27)**	**17.0 (12.8–25.8)**	**16.0 (12.0–25.0)**
GGT (U/L)	**21.8 (14.3–37.6)**	**18.0 (13.0–28.0)**	**33.2 (20.3–61.0)**	**26.3 (17–49.7)**	**35.8 (22.8–66.0)**	**33.0 (20.4–63.0)**	**31.4 (20.3–57.3)**	**30.0 (19.0–59.0)**	**28.0 (17.7–53.3)**	**25.0 (16.0–47.5)**

Significant differences (*p* < 0.05) between the study groups are marked in bold print. ALT: alanine transferase; ApoA1: apolipoprotein A1; ApoB100: apolipoprotein B100; AST: aspartate aminotransferase; GGT: gamma-glutamyl transferase; HbA1c: haemoglobin A1c; HDL-C: high-density lipoprotein-cholesterol; HT: hypertensive; LDL-C: low-density lipoprotein cholesterol; nonHT: non-hypertensive; TC: total cholesterol; TG: triglyceride.

**Table 2:** Laboratory parameters of the study population based on the grade of hypertension (Grade 1: Systolic 140–159 mm Hg and/or diastolic 90–99 mm Hg. Grade 2: Systolic 160–179 mm Hg or greater and/or diastolic 100–109 mm Hg. Grade 3: Systolic 180 mm Hg or greater and/or diastolic 110 mm Hg or greater). Values are presented as mean ± standard deviation or median (lower quartile–upper quartile).

	Grade 1	Grade 2	Grade 3
n	99 601	43 283	41 990
TC (mmol/L)	5.08 ± 1.19	5.15 ± 1.15	5.15 ± 1.1
LDL-C (mmol/L)	3.06 ± 0.99	3.07 ± 0.99	3.04 ± 0.96
TG (mmol/L)	**1.41 (1.01–2.01)**	**1.48 (1.08–2.07)**	**1.54 (1.12–2.17)**
HDL-C (mmol/l)	1.36 ± 0.41	1.37 ± 0.41	1.36 ± 0.39
ApoB100 (g/L)	0.98 ± 0.28	0.99 ± 0.28	1.0 ± 0.28
ApoA1 (g/L)	1.52 ± 0.31	1.54 ± 0.34	1.53 ± 0.29
glucose (mmol/L)	**5.7 (5.1–6.6)**	**5.9 (5.3–7.0)**	**6.0 (5.4–7.2)**
HbA1c (%)	6.1 (5.6–7.3)	6.3 (5.7–7.4)	6.3 (5.7–7.5)
uric acid (µmol/L)	**313 ± 95.1**	**323 ± 94.4**	**333 ± 91.4**
urea (mmol/L)	**5.43 (4.36–6.95)**	**5.8 (4.68–7.5)**	**6.03 (4.9–7.9)**
creatinine (µmol/L)	**74.4 (62.5–89.0)**	**77.0 (65.0–92.9)**	**79.0 (66.4–97.3)**
AST (U/L)	22.0 (18.0–29.7)	22.5 (18.5–30.0)	22.5 (18.5–29.5)
ALT (U/L)	22.0 (16.0–32.0)	22.1 (16.4–32.5)	22.5 (16.7–32.3)
GGT (U/L)	**31.0 (19.3–57.5)**	**33.3 (21.0–60.3)**	**34.0 (21.5–61.3)**

Significant differences (*p* < 0.05) between the study groups are marked in bold print. ALT: alanine transferase; ApoA1: apolipoprotein A1; ApoB100: apolipoprotein B100; AST: aspartate aminotransferase; GGT: gamma-glutamyl transferase; HbA1c: haemoglobin A1c; HDL-C: high-density lipoprotein-cholesterol; HT: hypertensive; LDL-C: low-density lipoprotein cholesterol; nonHT: non-hypertensive; TC: total cholesterol; TG: triglyceride.

## Data Availability

The data analysed in this study are subject to the following licenses/restrictions: The datasets generated and/or analysed during the current study are not publicly available due to privacy or ethical restrictions but are available from the corresponding author on reasonable request.

## Supplementary Materials

The following supporting information can be downloaded at: https://www.mdpi.com/article/10.3390/jcdd10080345/s1, Figure S1: Flowchart of the participants included in our study. (n: number of participant). Figure S2: Prevalence (%) of concomitant diseases in the (A) hypertensive and in the (B) non-hypertensive patient populations in different age groups. CaVD: cardiovascular disease; CeVD: cerebrovascular disease, PAD: peripheral arterial disease, Ren: renal disease.
