# Supplementary material for: Assessment of Hypertensive Patients’ Complex Metabolic Status Using Data Mining Methods
Source: J Cardiovasc Dev Dis. 2023 Aug 13;10(8):345. doi: 10.3390/jcdd10080345 (PMC10455679; doi:10.3390/jcdd10080345)
Supplement: Supplementary file 1 [file jcdd-10-00345-s001.zip › jcdd-2515469-supplementary.pdf]

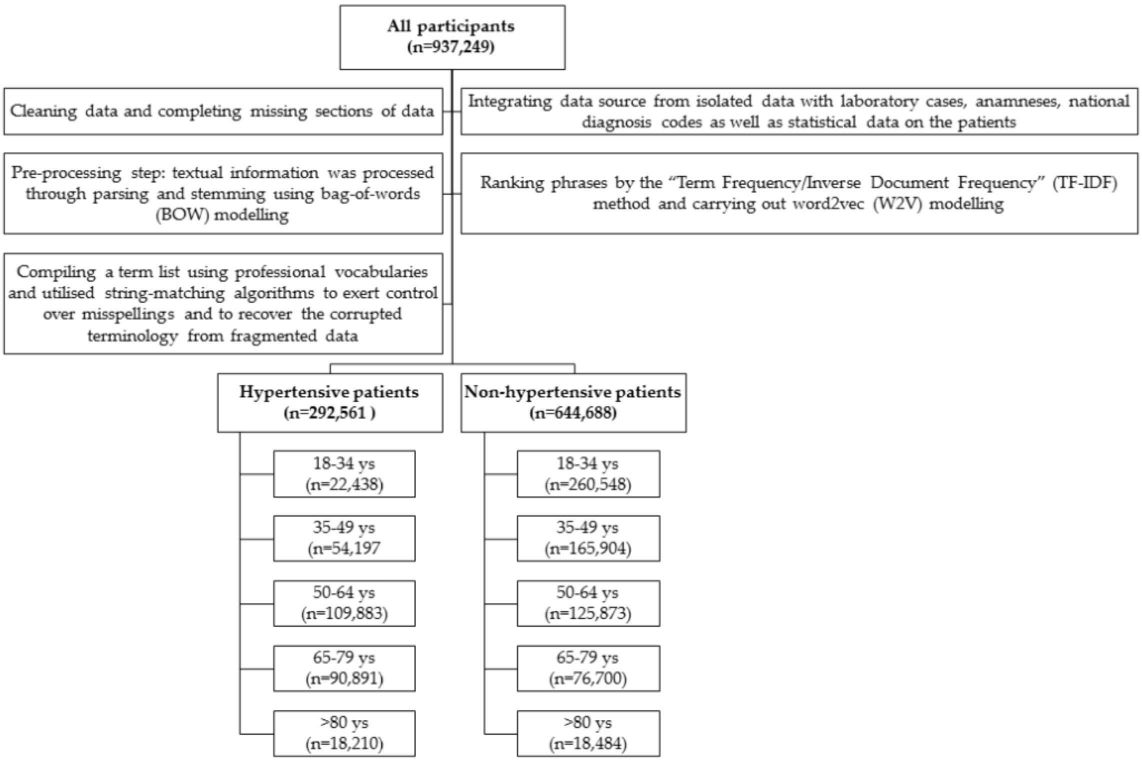

Figure S1: Flowchart of the participants included in our study. (n: number of participant)

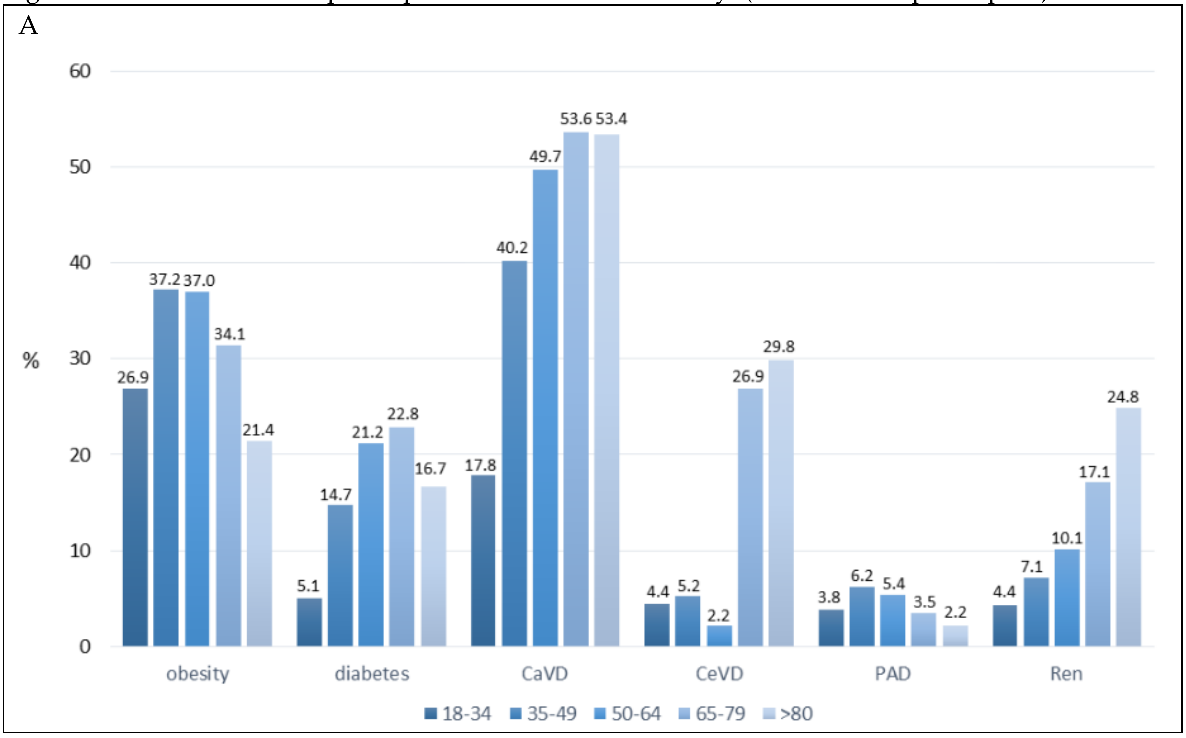

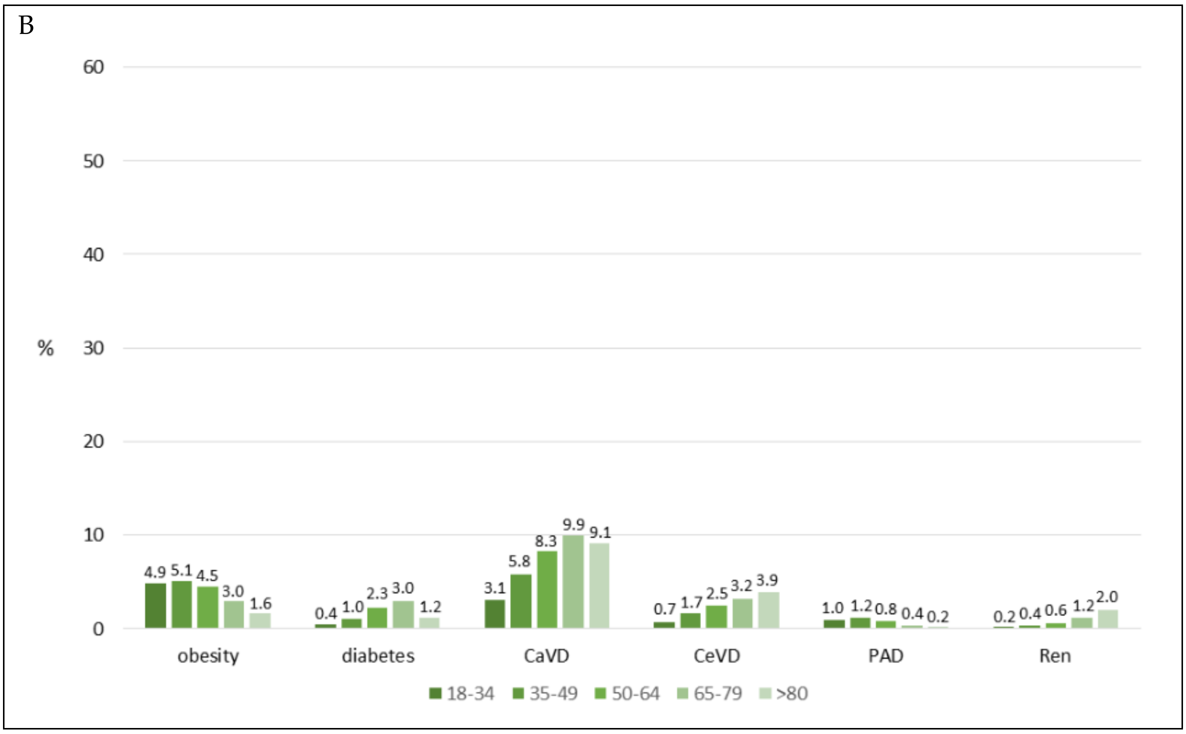

Figure S2: Prevalence (%) of concomitant diseases in the (**A**) hypertensive and in the (**B**) non-hypertensive patient populations in different age groups. CaVD: cardiovascular disease; CeVD: cerebrovascular disease, PAD: peripheral arterial disease, Ren: renal disease.
